# Exosome: A Novel Approach to Stimulate Bone Regeneration through Regulation of Osteogenesis and Angiogenesis

**DOI:** 10.3390/ijms17050712

**Published:** 2016-05-19

**Authors:** Yunhao Qin, Ruixin Sun, Chuanlong Wu, Lian Wang, Changqing Zhang

**Affiliations:** 1 Department of Orthopaedics, Shanghai Jiaotong University Affiliated Sixth People’s Hospital, Shanghai 200030, China; yunhao_qin@live.cn; 2 The Key Laboratory of Molecular Medicine, the Ministry of Education, Department of Biochemistry and Molecular Biology, Shanghai Medical College, Fudan University, Shanghai 200032, China; qingshuiqiuqian@126.com; 3 Shanghai Key Laboratory of Orthopaedic Implants, Department of Orthopaedics Surgery, Shanghai Ninth People’s Hospital, Shanghai Jiaotong University School of Medicine, Shanghai 200011, China; challengewu1988@163.com; 4 School of Medicine, Shanghai Tongji University School of Medicine, Shanghai 200092, China; dsv_231@hotmail.com

**Keywords:** exosome, osteogenesis, bone regeneration

## Abstract

The clinical need for effective bone regeneration therapy remains in huge demands. However, the current “gold standard” treatments of autologous and allogeneic bone grafts may result in various complications. Furthermore, safety considerations of biomaterials and cell-based treatment require further clarification. Therefore, developing new therapies with stronger osteogenic potential and a lower incidence of complications is worthwhile. Recently, exosomes, small vesicles of endocytic origin, have attracted attention in bone regeneration field. The vesicles travel between cells and deliver functional cargoes, such as proteins and RNAs, thereby regulating targeted cells differentiation, commitment, function, and proliferation. Much evidence has demonstrated the important roles of exosomes in osteogenesis both *in vitro* and *in vivo*. In this review, we summarize the properties, origins and biogenesis of exosomes, and the recent reports using exosomes to regulate osteogenesis and promote bone regeneration.

## 1. Introduction

In the clinic, lack of bone regeneration may result in a poor prognosis, even in common bone fractures. It has been reported that approximately 2% to 10% of bone fractures may develop non-union due to insufficient bone growth [1], and patients suffer from disabilities and may even have a shorter life expectancy as a result. The surgical removal of carcinomas is another major cause of insufficient bone regeneration, especially in patients with cancer-related bone metastasis [2]. Additionally, a higher incidence of obesity results in more pronounced musculoskeletal illnesses and decreased bone regeneration [3]. The ageing of the population exacerbates this situation. Aging not only results in a higher bone fracture risk due to loss of bone density but also diminishes the capability of bone regeneration [4]. Therefore, how to promote bone regeneration, especially in patients with large bone defects, remains a major challenge for the clinicians.

The current “gold standard” treatment in the clinical settings promotes bone regeneration through the use of autologous and allogeneic bone grafting. However, approximately 20%–30% of patients who undergo autologous bone grafts suffer from morbidity at the graft-harvesting site [5]. Moreover, autologous bone grafts cannot provide patients with large defects with sufficient bones [6]. After an allogeneic bone grafts, over 30% of patients suffer from complications, including fracture, non-union, and infection [7]. Allogeneic bone grafts may also result in graft-*versus*-host disease (GVHD) [8]. In addition, in cases in which patients received successful bone grafts, the recovery is time-consuming, up to one and half years [9]. Therefore, neither of these two options is the optimal, because they are expensive, uncomfortable for the patients, and have high risks of complication.

Biomaterials and the cell-based therapies are two major research fields in bone regeneration. However, there are some drawbacks to both treatments. The toxicity and immunogenicity of biomaterials may culminate in severe complications. Cell-based therapy is closely related to tumor and emboli formation [10]. Today, exosomes, ranging in size from 50–120 nm [11], with fewer safety considerations and powerful pro-osteogenesis abilities, provide researchers with a novel way to stimulate bone regeneration. This type of vesicles is endocytic origin and released by various cells and organs. Exosomes deliver various content [12], including DNAs, RNAs and proteins and are widely distributed, and are especially enriched in breast milk, semen, saliva, urine and sputum [13]. Exosomes can effectively stimulate regeneration in tissues and organs, including the heart, lung, liver and kidney. Small vesicles can also stimulate bone regeneration *in vitro* and *in vivo.* The good bone specificity and powerful bone regenerative properties make exosomes a potential treatment to enhance bone growth and to treat clinical bone diseases.

## 2. Bone Regeneration Requires the Coordination of Various Cells

Understanding bone biogenesis is a precondition for developing novel approaches to stimulate bone regeneration. Osteoblasts, osteoclasts and chondrocytes are the major cells involved in bone regeneration. The bone may regenerate in two different ways: by intramembranous ossification or endochondral ossification [14]. Intramembranous ossification gives rise to flat bones and endochondral ossification gives rise to long bone. Both ossifications begin with condensation of the mesenchyme and culminate in the formation of calcified bone. However, intramembranous ossification achieves bone calcification directly by mesenchymal stem cell (MSC) osteoblastic differentiation, whereas endochondral ossification incorporates a complicated step in which chondrocytes regulate the growth and formation of the skeleton [15]. During endochondral ossification, chondrocytes in the center of the cartilage stop proliferating and start to enlarge (hypertrophy), while synthesizing and releasing type X collagen [16]. Hypertrophic chondrocytes undergo mineralization and induce vessel penetration and osteoblast differentiation and migration. The recruited endothelial cells secrete vascular endothelial growth factor (VEGF) and direct chondroclasts to digest the surrounding matrix. Finally, hypertrophic chondrocytes undergo apoptotic cell death, and blood vessels and osteoblasts infiltrate the cartilage matrix and subsequently achieve bone growth and regeneration. 

In addition to osteoblasts, osteoclasts and chondrocytes, endothelial cells also have a large influence on bone regeneration. Endothelial cells stimulate osteoblast maturation and activity but inhibit the osteoblastic differentiation of osteoprogenitor cells [17]. Additionally, successful bone regeneration is closely related to successful angiogenesis, and impaired angiogenesis always results in failed bone regeneration. Inhibition of VEGF, a potent angiogenesis factor, results in the abnormal endochondral bone formation [18]. Endothelial cell-specific Notch knock-out not only impairs angiogenesis but also reduces osteogenesis, bone length and bone mass [19,20]. All of this evidence indicates that endothelial cells play important roles in bone regeneration.

Previous bone regeneration studies have mainly focused on stimulating the function of cells, and cell-to-cell communication has not been well studied. However, previous research has shown that cell-to-cell communication also greatly affects bone regeneration. Therefore, a therapy that could greatly increase osteogenic cell bone formation and the interaction between cells would be a potential future treatment. Exosomes, nanoscale vesicles ranging from 50–120 nm [11], have both properties, which has prompted intensive investigation of the exosomes. 

## 3. The Properties of Exosomes

**Figure 1:**
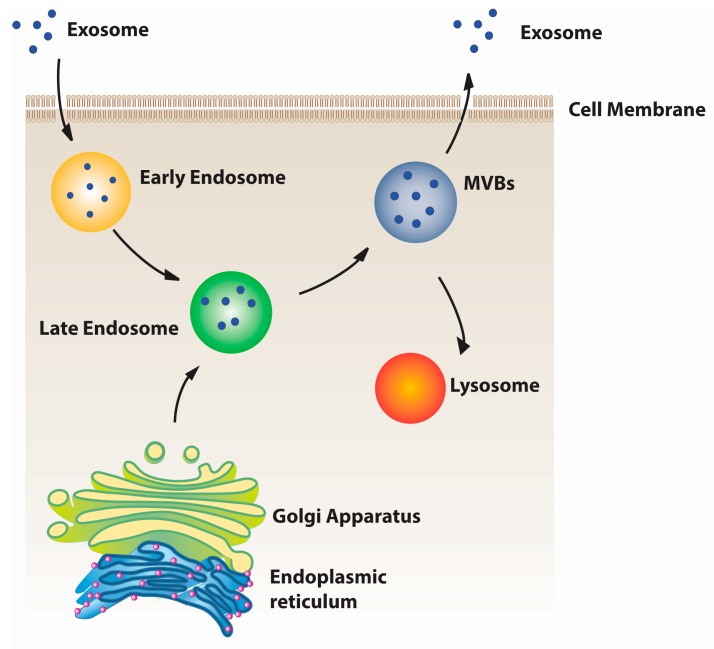
Exosome biogenesis: exosomes are generated in two distinct ways: the endocytic pathway and the biosynthetic pathway. The endocytic pathway begins by receiving extrinsic or intrinsic signals from the local milieu. Then, the plasma membrane begins to invaginate, and the early endosome subsequently forms. The early endosome becomes the late endosome under the regulation of multiple cell signaling pathways. The Golgi Apparatus and the endoplasmic reticulum also participate in exosome secretion. The multivesicle body resulting from the late endosome fuses with the plasma membrane and releases the exosome, or undergoes degradation.

Exosomes were first obtained from cell lines and described in 1981 [21], as exfoliated vesicles with ectoenzyme activities. The current definition is that they are membrane vesicles of endocytic origin [22] and are released into the extracellular environment upon the fusion with the plasma membrane (Figure 1) [23]. Exosomes are nanoscale vesicles ranging in size from 50–120 nm [11], with a density in sucrose of 1.13–1.19 g/mL and are wildly distributed both *in vivo* and *in vitro* [24] (Table 1). Many cells, including reticulocytes [24], dendritic cells [25], B cells [26], T cells [27], mast cells [28], epithelial cells [29] and tumor cells [30], secrete exosomes. Exosomes transport coding RNA [31], noncoding RNA [32], proteins [33], antigen presentation molecules [34], and DNA [35] between cells. By conveying proteins and RNAs, exosomes modulate the recipient cells and other organs function, activity, and commitment of recipient cells and other organs over a long distance [36]. 

Although how exosomes participate in cell-to-cell communication is not fully understood, several studies have revealed that ligand–receptor interaction plays an important role in this process [37]. This hypothesis was first proposed by Raposo, *et al.* [26], who noted that exosomes from B cells incorporate and transport functional antigen-presenting complexes. This discovery suggests that the mechanism of exosome participation in cell-to-cell communication involves receptor–ligand interactions [38]. The vesicles attach or fuse with the target cell membrane via exosome surface proteins such as Alix or Tumor Susceptibility 101 (TSG101), and tetraspanins such as CD9, CD63, CD81 and CD82 [39,40].

Although the cargoes transported by exosomes are diverse, exosomal proteins and RNAs are believed to play important roles in regulating the function of recipient cells [41]. Spectrometry data for exosomes have identified over 4000 different proteins in exosomes [11]. Though exosomal proteins differ substantially according to the origin of the exosome and have different functions, some proteins are shared by all types of exosomes [42]. These commonly-shared proteins are cell-to-cell communication related. For example, heat shock protein (HSP) 70 and HSP90 are shared by all exosomes and are key to protein trafficking [43]. Annexin is a membrane trafficking protein that is involved in fusion events and is enriched in exosomes. Additionally, the cytoskeletal proteins, including myosin, actin and tubulin, are found in exosomes. Regarding another potential functional content in exosomes, small RNAs have attracted the most attention [44,45]. Koppers-Lalic *et al.* [41] have provided a review of exosomal RNAs and have noted that the functional exosomal RNAs are critical in the regulation of cell commitment, differentiation, and activity. 

## 4. Exosomes Promote Regeneration in Various Tissues though Functional Cargo Transportation

The regenerative effect of exosomes has been validated in other tissues and organs, including the heart [37], lung [46], kidney [47] and brain [48]. Organ functions benefit from exosome treatment. In myocardial infraction mouse models, ventricular remolding and the left ventricular ejection fraction have been found to significantly improved after exosome treatment [37]. Exosomes also mediate cell function, thus, promoting regeneration. In hypoxia-induced pulmonary hypertension mice, exosomes treatment inhibits disease progression and protected the lung from hypertension through a MSC cytoprotective action. Furthermore, exosomes not only prevent apoptosis but also strengthen cell endurance. The renal injury is less severe, and exosome treatment improves renal function in mice with acute kidney injury [47]. In addition, modification of exosomes may provide a more effective treatment of diseases. Alvarez-Ervitl *et al.* [48] have demonstrated amelioration of Alzheimer’s disease through the injection of exosomes secreted from modified cells. Basu *et al.* [49] have reviewed of current exosomal treatment in neuroregeneration and skin regeneration. 

These studies have provided a foundation for exosome treatment and the exploration of the roles of exosomes in bone regeneration. Recent studies have demonstrated that exosome treatment stimulates bone regeneration *in vivo* and *in vitro*. Although the outcome of exosome treatment is inspiring, the exact underlying mechanism remains elusive. 

## 5. Exosomes Regulate Mesenchymal Stem Cell Osteogenic Differentiation

Exosomes directly regulate and guide MSCs into the osteoblastic lineage. MSC-derived exosomes can be used as biomimetic tools to induce naïve stem cells into to a osteogenic linage [50]. Profiling data for the MSC-derived exosome have revealed that nine miRNAs (let-7a, miR-199b, miR-218, miR-148a, miR-135b, miR-203, miR-219, miR-299-5p and miR-302b) are up-regulated and four miRNAs (miR-221, miR-155, miR-885-5p, miR-181a and miR-320c) are down-regulated during the process of MSC osteoblastic differentiation [51]. All of these miRNAs have roles in osteoblast function and activity. This profiling provides preconditions for further investigation and application of MSC-derived exosomes. However, the osteoblast itself also secretes exosomes, thus establishing a positive feedback of bone growth. Mineralizing osteoblast-derived exosome greatly increases osteoblastic differentiation related miRNAs, and activate Wnt signaling via Axin1 inhibition, thereby promoting MSC osteogenic differentiation [52]. Furthermore, eukaryotic initiation factor 2 in osteoblast-derived exosomes may also induce MSC to differentiate into osteoblast [53]. As is well known, the immune system and the hematopoietic system have a strong influence on bone growth, although the exact mechanism remains elusive. Exosomes may contribute to this process. Studies have demonstrated that dendritic cell- [54] and monocyte [55] cell-derived exosomes significantly stimulate MSC osteogenic differentiation *in vitro* by delivery of exosomal miRNAs (Table 2). MSC osteogenic differentiation is under the regulation of exosomes; however, which cell type-derived exosome is the most potent regulator and how the exosomes mediate MSC differentiation remain to be investigated.

## 6. Exosomes Regulate Osteoblast Proliferation and Activity

It is known that 4%–6% of the total resident cells in the bone are osteoblasts, whose major function is bone formation [56]. During bone formation, osteoblasts produce calcium- and phosphate-based minerals to form mineralized bone. Exosomes can also stimulate bone regeneration by directly regulating osteoblast proliferation and activity. Prostate cancer cell-derived exosomes increase human osteoblast proliferation by 1.5-fold [57], whereas matrix-derived exosome-treated osteoblasts generate more calcium deposits and greater ALP activity [58]. Prostate cancer cell-derived exosomes showed an excellent bone affinity [57]. Most injected PKH2-labeled exosomes enter the lung and the bone marrow within 24 h, and little is found in other organs. Whether other cell-type-derived exosomes share the same distribution remains unknown. The *in vivo* influence of exosomes on osteoblasts is also significant. Rats with calvarial defects benefit from bone marrow stromal cell-derived exosomes and show an earlier healing of defects [59] (Table 2). The exosomal miRNA-196a is the key factor stimulating the proliferation and activity of osteoblasts. Although both *in vivo* and *in vitro* studies underscore the importance of exosomes to osteoblast, more information is needed regarding the exact exosome treatment efficacy in bone systems.

## 7. Exosomes Regulate Osteoclast Maturation and Activity

As is well known, bone metastasis is closely related to the abnormal activation of osteoclasts. Research has shown that tumor cells induce osteolysis by secreting vesicles to increase the number and activity of mature osteoclasts. For example, multiple myeloma-derived exosomes internalized by the Raw264.7 cell lines and human primary osteoclast, increased expression of osteoclast marker, including Cathepsin K, Metalloproteinases 9, and Tartrate-resistant Acid Phosphatase (TRAP), thus promoting the maturation of osteoclasts [60]. In prostate cancer cell-derived exosomes, the vesicles also increase osteoclastogenesis by stimulating receptor activator of nuclear factor κB (RANK) expression [57]. 

In fact, the bone system itself is the most important regulator of osteoclast differentiation. Osteoblast- and osteocyte-derived lysosomal membrane protein 1 (LAMP1) positive exosomes also contain TRAP, RANK ligand, and osteoprotegerin (OPG), which are critical to osteoclast differentiation [61]. However, mature osteoclasts may regulate the cells themselves through exosome secretion. The profiles of osteoclast-derived exosomes indicate that RANK is highly enriched. The depletion of RANK-enriched exosomes results in inhibition of osteoclast formation [62] (Table 2). The roles of exosomes in osteoclasts may provide hints as to how bone formation and absorption are orchestrated.

## 8. Exosomes Are Potent Pro-Angiogenic Factors

Although there are no direct studies of the angiogenic ability of exosomes in bone, exosomes stimulate angiogenesis in other tissues and organs. The potent exosomal angiogenic ability may possibly stimulate bone growth and regeneration by increasing vessel formation. It has been demonstrated that exosomes stimulate endothelial cell proliferation, migration, and tube formation *in vitro,* such as placental MSC-derived exosomes [63]. Furthermore, exosomes also increase endothelial cell migration and tube formation through transportation of functional enzymes, including subunit of NADH oxidase [64], metalloproteinases and extracellular matrix metalloproteinase inducer [65]. Moreover, exosomes promote endothelial cell proliferation and vessel formation through exosomal miR-129, miR-136 [66] and the miR-17-92 cluster [63]. Not only do exosomes show angiogenic potency *in vitro*, but they also enhance angiogenesis *in vivo.* Research has shown that MSC-derived exosomes successfully improved angiogenesis in different animal models. For example, tail injection of MSC derived exosomes reduces myocardial ischemic/reperfusion injury and improves angiogenesis in the ischemic heart [67], and umbilical cord derived-MSC exosomes from human promotes blood perfusion and attenuated hind-limb ischemia [68] (Table 2). Exploring the roles that exosomes play in bone vessel formation is expected to lead to the development of novel treatments for bone regeneration. 

## 9. Advantages of Exosome Treatment

Exosome treatments have several advantages over the cell-based treatments. Living cell transplantation may cause more safety concerns than exosome treatment. Application of exosomes resolves toxicity and immunogenicity problems caused by biomaterial treatment, such as nanoparticles [10]. The vesicles can both positively and negatively regulate the immune response. MSC-derived exosomes keep the immune privileged properties of their origins. Patients presenting with intestinal graft-*versus*-host disease grade IV treated with MSC-derived exosomes undergo a significant amelioration of symptoms [69]. However, glioblastoma-derived exosomes activate an immune response to recognized glioblastoma cells. This advantage may greatly help researchers to develop novel immunotherapies [70]. Additionally, nonviable vesicles, compared with living cell transplantation, present a lower risk for severe complication, such as tumors, emboli formation, or GVHD. Furthermore, exosomes are very stable and can be kept approximately 6 months *in vitro* at −20 °C without loss of potency [71].

## 10. “Bench to Bedside”: Still a Long Way to Go

Several challenges prevent the development of exosomes into therapeutically agents. One major challenge is to achieve good manufacturing practices. Current exosomes isolation methods provide only a low exosome yield; for example, 5 × 10^6^ myeloma cells provide only 5–6 µg of exosomes [72]. Second, the method of exosome isolation is still controversial. There are two widely used and presumably accepted purification protocols, which use either repeated ultracentrifugation or ultrafiltration [73]. The drawback of these two protocols is the length of time required. Therefore, to develop a reliable method to isolate exosomes will greatly help future studies.

Another major concern is that the exact function of the genetic information that exosomes carry remains elusive. Thus, profiling exosomal contents is a precondition for clinical application. Currently, exosomal miRNAs are one of the major functional components of exosomes. The exosomes content varies according to different origins. Baglio *et al.* [74] have profiled the miRNA and tRNA information of bone marrow and adipose MSC. The miRNA expression is not significantly different between the cell types. However, the tRNAs show significant difference, especially for Sox2, POU5F1A/B, and Nanog. This finding indicates that the diverse cargoes in exosomes differ greatly according to the origin of the exosome. Furthermore, tumor-supportive miRNA and other bioactive factors are also found in MSC-derived exosomes [75]. Hence, profiling and understanding the exact function of exosomes is a precondition for clinical usage. 

Understanding the distribution of injected exosomes is important to control exosome location related side-effects. Studies showed that most exosomes go to bone and the lung; however, other studies have shown that exosomes may also enter the spleen, liver and kidney within the first 30 min after injection. Therefore, a clear investigation of distribution, dosage and clearance will be the foundation for assessing exosome safety [76].

## 11. Conclusions

In this report, we reviewed recent studies exploring the application of exosomes to regulate osteogenesis and angiogenesis. Although much preliminary data indicated that exosomes stimulate both osteogenesis and angiogenesis, the exact mechanism remains elusive. Before problems can be realized, reliable methods to identify and purify exosomes must first be developed. In addition, a better understanding of the roles that exosomes play in regulating osteogenesis and angiogenesis is also needed. Finally, which cell type- or tissue-derived exosome is the most potent regulator remains to be determined.

## Figures and Tables

**Table 1:** Exosome, microvesicle, apoptotic body: major similarities and differences.

Characteristic	Exosome	Microvesicle	Apoptotic Body
Size	50–120 nm	100–1000 nm	50–500 nm
Morphology	Cup-shaped	Heterogeneous	Heterogeneous
Protein Marker	Alix, Tsg101, CD63, CD9	Selectins, integrins, CD40	Histones
Origin	Multivesicular Body	Plasma Membrane	Programmed cell death
Mechanism of discharge	Exocytosis of MVBs	Budding from plasma membrane	Cell shrinkage and death
Composition	Protein, miRNA, mRNA	Protein, miRNA, mRNA	Protein, DNA, miRNA, mRNA

**Table 2:** Reported roles of exosomes in osteogenesis and angiogenesis.

Origin of Exosomes	Content Profile	*In Vitro* Effect	*In Vivo* Effect
MSCs [50]	Not mentioned	Induce osteogenesis differentiation in naive stem cells	No *in vivo* data
Mineralizing osteoblasts [52]	Axin1 inhibitor	Promote osteoblastic differentiation by activating Wnt signaling	No *in vivo* data
Osteoblasts [53]	Tumor susceptibility gene 101, flotillin 1 and 1069 other proteins	Promote osteoblastic differentiation by activating eukaryotic initiation factor 2	No *in vivo* data
Monocytes [55]	Small RNAs are enriched in exosomes	Runt-Related Transcript Factor 2, Osteocalcin and Bone Morphogenetic Protein 2 were Up-regulated in bone mesenchymal stem cells	No *in vivo* data
Prostate cancer cells [57]	miR-148a, miR-125a	Increased osteoblast proliferation	Most PKH2 labeled exosomes go to lung and bone marrow in 24 h. (liver, spleen, kidney, heart, thymus, brain, prostate)
Matrix [58]	Not mentioned	Increase Alkaline Phosphatase activity of osteoblast; Increase mineral deposition	No *in vivo* data
Bone MSCs [59]	miR-196a	Increased osteoblast activity	Stimulate bone growth in calvarial bone defect models
Myeloma cells [60]	Not mentioned	Induce pre-osteoclast maturation and migration	No *in vivo* data
		Promote osteoclast differentiation	
Osteoclasts [62]	RANK	Induce osteoclast differentiation	No *in vivo* data
Placental MSCs [63]	157 proteins enriched.	Increase endothelial cell migration, tube formation	No *in vivo* data
Platelets [64]	P22phox and gp91phox subunit of NADPH oxidase	Stimulate mRNA expression for angiogenic factors: Matrix metallopeptidase 9, vascular endothelial growth factor, interleukin-8, hepatocyte growth factor in endothelial cells	No *in vivo* data
Myocardial progenitor cells [65]	Metalloproteinases, extracellular matrix metalloproteinase inducer	Increase endothelial cell migration	No *in vivo* data
Bone marrow derived-stem cells [66]	miR-126, miR-139	Increase endothelial cell viability, proliferation and tube formation	No *in vivo* data
MSCs [67]	Not mentioned	Increase endothelial cells proliferation, migration and tube formation	Reduce myocardial ischemic/reperfusion injury; Improve angiogenesis in ischemic heart
Human umbilical cord derived MSCs [68]	Not mentioned	Increase endothelial cells proliferation, network formation. Significantly increased blood flow in ischemic model	Promote blood perfusion and attenuate hind-limb ischemia
Human induced pluripotent stem cell derived MSCs [77]	Not mentioned	Increase endothelial cell migration, proliferation, and tube formation	Promote blood perfusion and attenuate severe hind-limb ischemia
Chronic myeloid leukemia cells [78]	Not mentioned	Increase endothelial cell migration and tube formation	Promote matrigel induced tube formation in nude mice
Myelogenous leukemia [79]		Increase endothelial cell motility, ingrowth and vascularization	
Leukemia cells [80]	miR-17-92 cluster	Increase endothelial cell migration, proliferation and vessel formation	No *in vivo* data
Adipose MSC [81]	Artemin, Axl, Milk Fat Globule-EGF Factor-8, Oncostatin M, Stem Cell Factor, and thrombopoietin are enriched.	Increase vessel-like formation	Promote vessel formation in subcutaneous gel
